# Transactions of the Texas State Medical Association

**Published:** 1876-09

**Authors:** 


TRANSACTIONS OF THE TEXAS STATE MEDICAL ASSOCIATION.—Since the last issue of the JOURNAL, we have received the Transactions of the Texas State Medical Association for the years 1874 and 1875. The volume for 1874 contains a partial report of the meetings of 1872 and 1873, with a few of the papers presented at these meetings. In this volume of 200 pages we find a number of interesting and instructive papers, which we would be pleased to review did our space permit.

The volume for 1875 shows a decided improvement in the interest displayed by the physicians of the State. We are happy to be able to say that our brothers of the "Lone Star State" are not a whit behind the profession in the older States, presenting a volume in every way as creditable as in those States where the State associations have been organized and in good working order for the past quarter of a century. They are certainly alive to the importance of their organization, and if they continue their labors with the same interest, we predict that the Medical Association of Texas will in a few years occupy an advanced position.

We were happy to see that our old friend and confrere, Dr. H. W. Brown, formerly of Atlanta, Georgia, but now of Waco, Texas, was made President of the Association. We fully intended to have been present at the last annual meeting, but was prevented by illness. It is our intention to be present at their next annual meeting, which, if we mistake not, comes off in April, 1877, at Galveston.

